# Emergence of *Klebsiella pneumoniae* ST14 co-*harboring bla*_NDM-1_, *bla*_OXA-232_*, mcr*-1.1, and a novel IncI1 *tet*(X4) plasmid, with evidence of ColKP3 mobilization under antibiotic pressure

**DOI:** 10.1016/j.crmicr.2025.100466

**Published:** 2025-08-28

**Authors:** Thanawat Phuadraksa, Yanisa Choominthong, Sineewanlaya Wichit, Sakda Yainoy

**Affiliations:** Department of Clinical Microbiology and Applied Technology, Faculty of Medical Technology, Mahidol University, Thailand

**Keywords:** *Klebsiella pneumoniae* ST14, Carbapenem-resistant enterobacterales (CRE), *tet*(X4), *mcr*-1, One Health

## Abstract

Companion animals and environmental niches act as interconnected reservoirs of antimicrobial resistance (AMR) genes, facilitating their persistence and horizontal transfer across hosts and ecosystems. Pet-associated environments, within the One Health framework linking human, animal, and environmental health, remain an underrecognized source of AMR dissemination. Pet grooming facilities generate wastewater containing bacteria from animal skin, fecal matter, and contaminated surfaces, potentially acting as factors that facilitate environmental contamination and zoonotic transmission. Here, we describe the isolation and complete genomic characterization of an extensively drug-resistant *Klebsiella pneumoniae* strain from wastewater at a pet grooming facility in Bangkok, Thailand. Whole-genome sequencing identified the isolate as sequence type (ST) 14, a globally disseminated high-risk clone associated with multidrug resistance and clinical outbreaks. The strain harbored four clinically significant resistance genes, *bla*_NDM-1_, *bla*_OXA-232_, *mcr*-1.1, and *tet*(X4), each located on distinct plasmids. To our knowledge, this is the first report of *tet*(X4) in *K. pneumoniae* ST14. The gene was found on a novel IncI1-type plasmid with a composite transposon, suggesting recent acquisition through horizontal gene transfer. Conjugation assays confirmed high transfer efficiency and phenotypic tigecycline resistance. In this study, although *bla*_OXA-232_ was carried on a non-conjugative ColKP3-type plasmid, colistin selection facilitated its transfer with plasmid size expansion, indicating antibiotic-driven mobilization. These findings highlight the evolutionary adaptability of *K. pneumoniae* ST14 and the risk posed by pet-associated wastewater as a reservoir for clinically important AMR genes. Integrated genomic surveillance and targeted One Health interventions are urgently needed to prevent environmental and zoonotic spread.

## Highlights

• XDR *K. pneumoniae* ST14 was isolated from pet grooming wastewater in Bangkok.
• Strain harbored *bla*_NDM-1_, *bla*_OXA-232_, *mcr*-1.1, *tet*(X4) on distinct plasmids (1st report).
• *tet*(X4) was on a novel *IncI1* plasmid with a unique transposon, suggesting recent transfer.
• Colistin promoted co-transfer of *ColKP3: bla*_OXA-232,_ a non-conjugative plasmid.
• Highlights ST14 adaptability and need for One Health AMR surveillance in the environment.

## Graphical abstract

**Figure:**
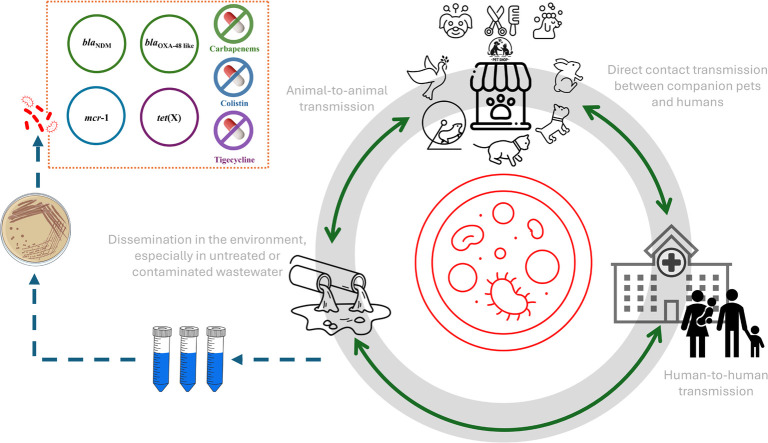

## 1 Introduction

Antimicrobial resistance (AMR) is a growing public health threat that affects human, animal, and environmental health. In recognition of this threat, the World Health Organization (WHO) released its 2024 priority list of bacterial pathogens, identifying carbapenem-resistant Enterobacterales (CRE) and carbapenem-resistant *Acinetobacter baumannii* as “Critical” priority pathogens (Organization, 2024). The primary mechanism driving carbapenem resistance in Enterobacterales is the acquisition of carbapenemase genes, such as *bla*_KPC_, *bla*_NDM_, *bla*_OXA-48_-like, *bla*_IMP_, and *bla*_VIM_, which are often mediated by horizontal gene transfer through mobile genetic elements and plasmids (Caliskan‑Aydogan and Alocilja, 2023). Due to the limited treatment options for CRE infections, last-resort antibiotics such as colistin and tigecycline have been widely used (Chirabhundhu et al., 2024, Petrosillo et al., 2019). However, the emergence of resistance to these agents, especially through mobile resistance determinants, has raised additional concern. Colistin is a cationic antimicrobial peptide that targets the bacterial outer membrane by binding to lipid A of lipopolysaccharides, causing membrane disruption. Its widespread use in both human medicine and livestock production has led to the emergence of plasmid-mediated resistance genes (Liu et al., 2016). To date, ten mobilized colistin resistance (*mcr*) genes have been identified (Hussein et al., 2021). Among them, *mcr-*1 is the most prevalent globally and is most commonly found in *Escherichia coli, Klebsiella* spp., and *Salmonella* spp. (Mmatli et al., 2022). Tigecycline, a glycylcycline antibiotic derived from minocycline, is effective against a broad range of multidrug-resistant Gram-negative and Gram-positive bacteria by inhibiting bacterial protein synthesis (Yaghoubi et al., 2022). However, resistance to tigecycline has also emerged. It is primarily driven by two mobile genetic mechanisms, including enzymatic inactivation via *tet*(X) variants (He et al., 2019) and active efflux via the *tmexCD-toprJ* efflux pump (Lv et al., 2020). *tet*(X) genes encode flavin-dependent monooxygenases capable of degrading tigecycline and other tetracyclines. The most concerning variants are *tet*(X3), *tet*(X4), and *tet*(X5), which have been reported mainly in China and other regions of East Asia (Chen et al., 2020; He et al., 2019). The *tmexCD-toprJ* operon, a plasmid-encoded resistance-nodulation-division (RND) efflux pump, also contributes to high-level tigecycline resistance. Multiple variants (*tmexCD1–4-toprJ1–4*) have been described across diverse Gram-negative species isolated from animals, humans, and the environmental sources (Dong et al., 2022; Long et al., 2024). The convergence of resistance determinants to carbapenems, colistin, and tigecycline within a single bacterial strain is particularly concerning, as it severely restricts treatment options. Such co-resistance profiles have been reported in clinical isolates (Fang et al., 2025), livestock (Lu et al., 2022; Tang et al., 2021), companion animals (Khine et al., 2024), and environmental sources (Li et al., 2024; Zhu et al., 2023). For example, isolates co-harboring *bla*_NDM-5_, *mcr*-1, and *tet*(X4) have been detected in *E. coli* from pigs in China (Lu et al., 2022). Similarly, *A. veronii* carrying *bla*_KPC-2_, *mcr*-3.17, and *tmexCD-toprJ* variants have been isolated from hospital sewage (Zhu et al., 2023). Plasmid-mediated co-transfer of *mcr*-8, *bla*_NDM-1_, and *tmexCD-toprJ* in *K. pneumoniae* further highlights the potential for accumulation of resistance genes within single mobile elements (Wang et al., 2022). Additionally, recent reports of related resistant strains in both humans and companion animals suggest the possibility of interspecies transmission (Khine et al., 2024). Environmental reservoirs, particularly wastewater systems, serve as hotspots for AMR gene persistence and exchange. These systems often receive effluents from healthcare facilities, households, and animal care settings, facilitating the intersection of diverse bacterial communities and resistance elements (Berendonk et al., 2015; Hendriksen et al., 2019). In Thailand, AMR genes have been widely detected in domestic, hospital, and agricultural wastewater, emphasizing the importance of environmental monitoring as a component of the One Health approach (Thamlikitkul et al., 2019).

In this study, we investigated wastewater collected from 47 pet grooming facilities in Bangkok and Nakhon Pathom, Thailand, to assess the presence of Gram-negative bacteria harboring resistance determinants to carbapenems, colistin, and tigecycline. Phenotypic and genotypic screening revealed one isolate co-carrying *bla*_NDM_, *bla*_OXA-48-like_, *mcr*-1, and *tet*(X4). Whole-genome sequencing identified this strain as *Klebsiella pneumoniae* sequence type (ST) 14, a high-risk clone previously associated with the dissemination of carbapenemase genes in clinical settings. In addition, we observed the co-transfer of ColKP3, a non-conjugative plasmid carrying *bla*_OXA-232_ (a *bla*_OXA-48-like_ gene), along with an *mcr*-1.1 harboring plasmid. This co-transfer event has not been previously reported. To the best of our knowledge, this is the first report of an ST14 *K. pneumoniae* isolate co-harboring *bla*_NDM-1_, *bla*_OXA-232_, *mcr*-1.1, and *tet*(X4). These findings underscore the role of animal-associated environmental niches in the emergence and spread of extensively drug-resistant bacteria. This further highlights the urgent need for integrated surveillance frameworks that encompass human, animal, and environmental health under the One Health paradigm.

## 2 Materials and methods

### 2.1 Sample collection and bacterial isolation

A total of 141 wastewater samples (50 mL each) were collected between 2023 and 2024 from 47 pet grooming facilities and/or their surrounding drainage areas in Bangkok and Nakhon Pathom, Thailand. For each shop, three samples were collected. Each sample was transported on ice and processed within 4 h of collection. Samples were centrifuged at 15,000 rpm for 10 min, and the resulting pellets were resuspended in 5 mL of sterile distilled water. The suspensions were then inoculated into 100 mL of Luria-Bertani (LB) broth and incubated with shaking at 37 °C for 15 h. Following enrichment, each culture was serially diluted (1:100 to 1:10,000), and plated onto CHROMagar supplemented with colistin (2 mg/L), tigecycline (2 mg/L), or ertapenem (2 mg/L), as well as combinations of two or all three antibiotics, to enable selective isolation. Bacterial isolates were identified using matrix-assisted laser desorption/ionization time-of-flight mass spectrometry (MALDI-TOF MS; Biotyper system, Bruker Daltonik, Leipzig, Germany) according to the manufacturer's protocol. The presence of antimicrobial resistance genes, including *mcr*-1 to *mcr*-10, *tet*(X), and the major five carbapenemase genes (*bla*_KPC_, *bla*_NDM_, *bla*_OXA48_-like, *bla*_IMP_ and *bla*_VIM_), was screened by multiplex PCR according to the previously established protocols (Hsieh et al., 2021; Phuadraksa et al., 2022a; Poirel et al., 2011; Tullayaprayouch et al., 2025).

### 2.2 Antimicrobial susceptibility testing

Antimicrobial susceptibility to 15 antibiotics, including amikacin, cefiderocol, cefepime, cefotaxime, cefoxitin, ceftazidime, ciprofloxacin, chloramphenicol, colistin, ertapenem, gentamicin, imipenem, meropenem, tetracycline, and tigecycline, was examined. The minimum inhibitory concentrations (MICs) of all antibiotics was determined using the broth microdilution method (BMD), as recommended by the Clinical and Laboratory Standards Institute (CLSI) (James S. Lewis II, 2024). *E. coli* ATCC 25922 and *P. aeruginosa* ATCC 27853 were used as the quality control strain. The results were interpreted according to the CLSI 34^th^ edition guidelines.

### 2.3 Plasmid conjugation

The transferability of plasmids carrying antibiotic resistance genes was assessed using a filter-mating conjugation assay, as previously described (Phuadraksa et al., 2022b). Briefly, *K. pneumoniae* strain KP_WW21, harboring *bla*_OXA-232_, *bla*_NDM-1,_ *mcr*-1.1, and *tet*(X4) was used as the donor, while *E. coli* J53, a sodium azide-resistant strain, was used as the recipient. Donor and recipient cells were mixed at a 1:2 ratio, applied to a membrane filter, and incubated on LB agar at 37 °C for 4 h. Transconjugants were selected on MacConkey agar supplemented with sodium azide (150 mg/L) and either colistin (2 mg/L), tigecycline (2 mg/L), ertapenem (2 mg/L), or combinations of two or all three antibiotics. The presence of transferred antibiotic resistance genes in the transconjugants was confirmed by PCR.

### 2.4 Whole-genome sequencing

The bacterial genomic DNA was extracted by DNeasy Ultraclean Microbial kit (Qiagen, CA, USA) according to the manufacturer’s instructions. The DNA samples were subsequently sequenced through NovaSeq 6000-PE150 platform (Illumina, San Diego, CA, USA) to generate paired-end 150-bp reads and Nanopore PromethION platform (Nanopore, Oxford, UK) in accordance with the Rapid Sequencing Kit. *De novo* hybrid assembly of Illumina short reads and PromethION long reads was performed using Unicycler v0.4.8 (Wick et al., 2017). The complete genome was then annotated through PROKKA and RAST server (Aziz et al., 2008; Seemann, 2014). Acquired antimicrobial resistant genes, plasmid replicons, and virulence genes were determined using Resfinder, PlasmidFinder (Carattoli and Hasman, 2020), and VirulenceFinder, respectively. Based on the seven house-keeping genes (*gapA, infB, mdh, pgi, phoE, rpoB and tonB*), the sequence type (ST) was identified using Institut Pasteur server. Furthermore, the phylogenetic tree was performed and visualized through Roary (Page et al., 2015) and iTOL (Letunic and Bork, 2021), respectively. Moreover, the pairwise single nucleotide polymorphisms (SNPs) distances were generated by snp-dists v0.8. In addition, plasmid visualization and comparison were performed using Proksee (Grant et al., 2023).

## 3 Results

### 3.1 Bacterial isolation and identification

In this study, a carbapenem-, colistin-, and tigecycline-resistant *K. pneumoniae* strain, KP_WW21, was isolated from wastewater collected from a pet grooming shop in Bangkok, Thailand. Screening for the presence of antibiotic resistance genes by multiplex PCR showed that KP_WW21 harbored *bla*_NDM_, *bla*_OXA-48_-like, *mcr*-1, and *tet*(X) genes. Confirmation of the presence and variant identity of these genes by Sanger DNA sequencing revealed that *bla*_NDM_, *bla*_OXA-48_-like, *mcr*-1, and *tet*(X) in KP_WW21 were 100 % identical to the reference sequences of *bla*_NDM-1_ (accession no NG_049326.1), *bla*_OXA-232_ (accession no NG_049528.1), *mcr*-1.1 (accession no NG_050417.1), and *tet*(X4) (accession no NG_065852.1), respectively.

### 3.2 Occurrence of *bla*_NDM-1_, *bla*_OXA-232_, *mcr*-1, and *tet*(X4)-harboring *K. pneumoniae* ST14

The complete genome of *K. pneumoniae* KP_WW21 was sequenced by hybrid WGS approach. KP_WW21 consists of a 5,362,511 bp chromosome and 5 plasmids (pKP_WW21-NDM, pKP_WW21-mcr, pKP_WW21-tetX, pKP_WW21-OXA, and pKP_WW21–5), ranging from 6,141 to 307,479 bp (Table S1). The strain KP_WW21 was then confirmed as *K. pneumoniae* using KmerFinder. Antimicrobial susceptibility testing revealed that the isolate was resistant to nearly all of the tested antimicrobial agents, including gentamicin, amikacin, ciprofloxacin, ceftazidime, cefotaxime, cefoxitin, cefepime, ertapenem, imipenem, meropenem, colistin, tetracycline, and tigecycline. However, the isolate demonstrated intermediate to chloramphenicol and susceptibility to cefiderocol (Table 1).

**Table 1:** The minimal inhibitory concentrations (MICs) of bacterial isolates.

Isolate	AMR genes	Minimal Inhibitory Concentration; MICs (mg/L)														
		AK	FDC	FEP	CTX	FOX	CAZ	CIP	C	CL	ETP	GM	IPM	MEM	TE	TGC
KP_WW21	*mcr*-1.1, *tet*(X4), *bla*_NDM-1_, *bla*_OXA-232_	>16	2	>16	>16	>128	≥16	≥4	8	16	≥128	>16	≥128	≥128	>16	16
*E. coli* J53	-	4	≤0.03	≤1	≤0.25	≤8	0.5	≤0.25	4	≤0.25	≤0.25	2	2	≤0.25	2	0.5
(T) Clt clone 1	*mcr*-1.1, *bla*_OXA-232_	>16	≤0.03	≤1	≤1	≤8	>16	4	4	2	8	>16	8	8	2	0.5
(T) Clt clone 2	*mcr*-1.1, *tet*(X4)	≤1	≤0.03	≤1	≤1	≤8	≤1	≤0.25	4	2	≤0.25	≤1	2	≤0.25	>16	16
(T) TG	*tet*(X4)	≤1	≤0.03	≤1	≤1	≤8	≤1	≤0.25	4	≤0.25	≤0.25	≤1	2	≤0.25	>16	16
(T) Etp	*bla*_NDM-1_	>16	0.125	>16	>16	>128	>16	4	4	≤0.25	64	>16	32	32	>16	0.5
(T) Clt-Etp	*mcr*-1.1, *bla*_NDM-1_	>16	0.125	>16	>16	>128	>16	4	4	2	32	>16	16	16	2	0.5
(T) Etp-TG	*tet*(X4), *bla*_NDM-1_	>16	0.125	>16	>16	>128	>16	1	4	≤0.25	64	>16	32	32	>16	16
(T) Clt-TG	*mcr*-1.1, *tet*(X4)	≤1	≤0.03	≤1	≤1	≤8	≤1	≤0.25	4	2	≤0.25	≤1	2	≤0.25	>16	16

AK, amikacin; FDC, cefiderocol; FEP, cefepime; CTX, cefotaxime; FOX, cefoxitin; CAZ, ceftazidime; CIP, ciprofloxacin; C, chloramphenicol; CL, colistin; ETP, ertapenem; GM, gentamicin; IPM, imipenem; MEM, meropenem; TE, tetracycline; TGC, tigecycline; (T) Clt, transconjugant selected on medium containing colistin; (T) TG, transconjugant selected on medium containing tigecycline; (T) Etp, transconjugant selected on medium containing ertapenem; (T) Clt-Etp, transconjugant selected on medium containing colistin and ertapenem; (T) Clt-TG, transconjugant selected on medium containing colistin and tigecycline; (T) Etp-TG, transconjugant selected on medium containing ertapenem and tigecycline.

**Fig. 1:**
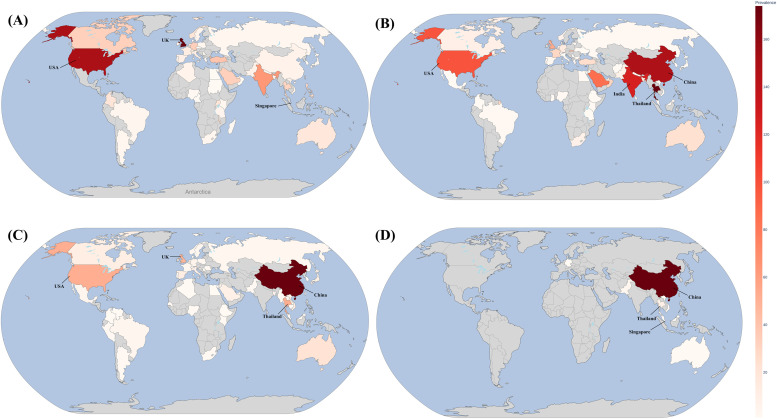
Global distribution of *K. pneumoniae* isolates carrying key resistance determinants. (A) sequence type 14, (B) *bla*_OXA-232_, (C) *mcr*-1, and (D) *tet*(X). The red gradient indicates the number of *K. pneumoniae* isolates reported from each country.

Virulence genes including genes encoding type I fimbriae (*fimA, fimB, fimC, fimD, fimE, fimF, fimG, fimH, fimI, fimK*), type III fimbriae (*mrkA, mrkB, mrkC, mrkD, mrkF, mrkJ, mrkI, mrkH*), capsule (*galF, gndA, rfbK1,ugd, rcsA, rcsB*), LPS (*rfbA, rfbB, rfbD*), yersiniabactin (*fyuA*/*psn, ybtE, ybtT, ybtU, irp1, irp2, ybtA, ybtP, ybtQ, ybtX, ybtS*), type VI secretion system (*sciN*/*tssJ, tssG, tssF, impA*/*tssA, icmF*/*tssM, vgrG*/*tssI, clpV*/*tssH, hcp*/*tssD, ompA, dotU*/*tssL, vasE*/*tssK, vipB*/*tssC, vipA*/*tssB*), enterobactin (*entA, entB, entC, entE, entF, entS, fepA, fepB, fepC, fepD, fepG, fes*), aerobactin (*iutA*), outer membrane protein A (*ompA*), feric uptake regulator (*fur*), acriflavine resistance protein (*acrA, acrB*), *E. coli* common pilus (*yagV*/*ecpE, yagW*/*ecpD, yagX*/*ecpC, yagY*/*ecpB, yagZ*/*ecpA, ykgK*/*ecpR*), siderophore esterase (*iroE*), and RNA polymerase sigma factor (*rpoS*) were identified through VirulenceFinder. In addition, Resfinder analysis was performed to identify antimicrobial-resistant genes. Among them, *aac(6′)-Ib-cr, bla*_CTX−__M-15_, *bla*_SHV-106_, *bla*_SHV-28_, *bla*_OXA-1_, *fosA6, catB3, OqxA, OqxB*, and *dfrA1* were located on the chromosome, while all others were located on plasmids. Moreover, plasmid replicons, including ColKP3, IncFIB, IncFIB, IncHI1B, IncI1, IncI2, and IncR were identified among these plasmids. In this study, multilocus sequence typing (MLST) analysis performed by PubMLST revealed that the sequence of KP_WW21 was identified as ST14. Based on *Klebsiella* PasteurMLST database, *K. pneumoniae* ST14 was commonly found in UK (174/1,003, 17.35 %), followed by USA (150/1,003, 14.96 %) and Singapore (73/1,003, 7.28 %). As shown in Fig. 1A, *K. pneumoniae* ST14 was disseminated across six continents around the world and found in various sources, including human, animal and environmental. The majority of isolates were recovered from human sources (879/1,001, 87.8 %), with significantly fewer from animals (11/1,001, 1.1 %) and environmental samples (1 %) (Table S2). Therefore, this clone could be transferred across origins and may serve as reservoirs for horizontal and vertical transmission of antibiotic-resistant genes.

### 3.3 Characterization of carbapenemase genes harboring plasmids

As shown in Fig. S1 and S2, the *bla*_NDM-1_ gene was located on a 307,479 bp IncFIB-IncHI1B hybrid plasmid (pKP_WW21-NDM; NCBI accession no CP192294). In contrast, the *bla*_OXA-232_ gene was carried on a much smaller, 6,141 bp ColKP3-type plasmid (pKP_WW21-OXA; NCBI accession no CP192298). A hybrid WGS revealed that pKP_WW21-OXA contained only a single antimicrobial resistance gene, *bla*_OXA-232_, which confers resistance to carbapenems. In contrast, pKP_WW21-NDM carried a wide array of resistance genes, including *aadA2, bla*_OXA-1_, *bla*_NDM-1_, *msr(E), aph(3′)-VI, armA, qnrB1, sul1, dfrA12*, and *dfrA14*. These genes confer resistance to multiple antibiotic classes, such as aminoglycosides, quinolones, beta-lactams, folate pathway antagonists, macrolides, streptogramin B, and amphenicols.

In the pKP_WW21-NDM, a total of 17 genes related to replication/recombination/repair, 4 phage-associated genes, 54 genes involved in integration and excision, 14 genes linked to stability/transfer/defense, and 18 genes specifically associated with transfer activity, were identified (Fig. S1). To explore the prevalence of *bla*_NDM_-positive *K. pneumoniae*, the location information of 15,771 isolates were retrieved from the NCBI database using the search criteria “AMR_genotypes: *bla*_NDM_” and “species_taxid:573” (Table S3). As shown in Fig. S3, the highest number of *bla*_NDM_ positive *K. pneumoniae* was presented by the USA (3,909/15,771, 24.79 %), followed by China (1,564/15,771, 9.92 %), Thailand (643/15,771, 4.07 %), and Bangladesh (602/15,771, 3.82 %). As shown in Fig. S1, The genetic context of *bla*_NDM-1_ on pKP_WW21-NDM included *ISAba125*-*ISEc33*-*bla*_NDM-1_-*ligA*-*ISAba125*, suggesting composite transposon. The presence of *ISEc33*, an insertion sequence associated with mobilization, further enhances the potential for horizontal gene transfer and plasmid dissemination among bacterial populations. The sequence of pKP_WW21-NDM was then blasted against the NCBI database and top 10 matches with query cover >30 % and >99 % identity were identified, these include KP617 plasmid KP-p1 (CP012754.1), PittNDM01 plasmid p1 (CP006799.1), AR_0068 plasmid unitig_1 (CP020068.1), pIncHI1B_DHQP1300920 (CP016921.1), NH34 plasmid pNH34.1 (CP034406.1), KPN528 plasmid pKPN528–1 (CP020854.1), CDC 0106 plasmid unitig_1 (CP022612.1), Nord77_R886 plasmid pR886_1 (CP091602.1), BA27935 plasmid pvirulence_VBA27935 (CP058799.1), and BA10835 plasmid pvirulence_VBA10835 (CP053766.1). Comparative analysis revealed a conserved backbone and surrounding regions of *bla*_NDM-1_ across these plasmids (Fig. S1). This high degree of conservation suggests that *bla*_NDM-1_ and its associated genetic elements are maintained within a stable and functionally optimized plasmid framework. This stability may enhance the persistence and spread of *bla*_NDM-1_ among various bacterial hosts. The transferability of pKP_WW21-NDM was assessed through conjugation experiments, which demonstrated a high transfer efficiency, 1.4 × 10^–3^ CFU per recipient cell. The transconjugant exhibited a >16-fold increase in the carbapenems MIC when compared with that of the recipeint cell (Table 1).

For pKP_WW21-OXA, a total of 1 gene associated with replication/recombination/repair, 1 gene classified under stability/transfer/defense, and 2 genes solely involved in transfer activity were identified (Fig. S2). To explore the prevalence of *bla*_OXA-232_-positive *K. pneumoniae*, the location information of 3,982 isolates were retrieved from the NCBI database using the search criteria “AMR_genotypes: *bla*_OXA-232_″ and “species_taxid:573” (Table S4). As shown in Fig. 1B, the highest number of *bla*_OXA-232_ positive *K. pneumoniae* was presented by Thailand (585/3,982, 14.69 %), followed by China (498/3,982, 12.51 %), India (422/3,982, 10.60 %), and the USA (330/3,982, 8.29 %). The sequence of pKP_WW21-OXA was then blasted against the NCBI database and top 10 matches with query cover 100 % and identity >99 % were identified, these included pJUNP053-OXA (LC507653.1), p RIVM_C017900_3 (CP068853.1), pRIVM_C018383_4 (CP068839.1), pKP151_OXA232 DNA (LC613148.1), pAI1235P (CP079128.1), NB5306 plasmid unnamed3 (CP034762.1), pRIVM_C018199_5 (CP068846.1), pRIVM_C017733_3 (CP068856.1), pKP4777–3 (CP186372.1), pK1–29 (MH523449.1) (Fig. S2). Moreover, the sequence was 99.8 % identical to the previously reported 6.1-kb *bla*_OXA-232_ harboring plasmid pkNICU5 isolated from a *K. pneumoniae* strain in China, with 100 % coverage suggesting stable dissemination (Yin et al., 2017). It was noted that *bla*_OXA-232_ on ColKP3 is a non-conjugative plasmid, and as such, it is incapable of being transferred (Potron et al., 2013; Yin et al., 2017). This aligns with our conjugation experiments, in which no transfer of *bla*_OXA-232_ was observed under selection with either ertapenem or tigecycline. However, when colistin was used for selection, transfer did occur, with a high transfer efficiency of 4.2 × 10^–3^ CFU per recipient cell. The transconjugant was subsequently sequenced through hybrid WGS, which revealed that the plasmid harboring *bla*_OXA-232_ was still on ColKP3 replicon type, but the size was up to 12,273 bp (acession no CP195071) (Fig. S4). The transconjugant exhibited a 4-fold increase in the imipenem MIC and >16-fold increase in other carbapenems when compared with that of the recipeint cell (Table 1). These results suggested that the *bla*_OXA-232_ gene on pKP_WW21-OXA can be transferred and expressed in transconjugant. These results suggest that the *bla*_OXA-232_ gene, located on pKP_WW21-OXA, was mobilized under colistin selection, indicating conditional transferability and functional expression in the transconjugant.

### 3.4 Characterization of *mcr*-1 harboring plasmid

The *mcr*-1.1 gene was located on a 61,805 bp IncI2-type plasmid (pKP_WW21-mcr; NCBI accession no CP192297) (Fig. S5A). This plasmid contained only one antimicrobial resistant gene, *mcr*-1.1. Genome annotation identified a total of 5 genes involved in replication/recombination/repair, 1 phage-associated gene, 2 genes related to integration/excision, 7 genes involved in stability/transfer/defense, and 24 genes associated exclusively with transfer functions. The abundance of mobility-related genes underscores the plasmid’s high potential for horizontal dissemination.

**Fig. 2:**
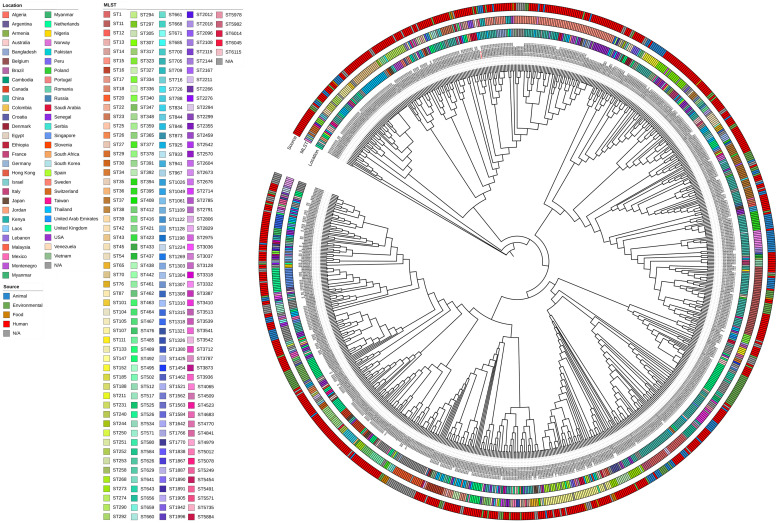
Phylogenetic tree of 859 *mcr*-positive *K. pneumoniae* isolates, with KP_WW21 from this study labeled in red. The tree was constructed using maximum likelihood analysis based on 1238 core genome SNPs. From the center outward, the colored rings represent the country of isolation, MLST type, and source of isolation, respectively.

To explore the prevalence of *mcr*-positive *K. pneumoniae*, genome sequences of 859 isolates were retrieved from the NCBI database using the search criteria “AMR_genotypes: *mcr*” and “species_taxid:573” (Table S5). Among the isolates with identifiable source information, China accounted for the largest proportion (240/859; 27.94 %), followed by Thailand (75/859, 8.73 %), the USA (73/859, 8.50 %), and the United Kingdom (68/859, 7.92 %), as illustrated in Fig. 1C. Host source analysis revealed that these isolates were derived from humans, animals, and environmental samples, underscoring the zoonotic and environmental reservoirs. MLST analysis revealed significant genetic diversity among the 859 *mcr*-positive *K. pneumoniae* isolates, which were assigned to numerous sequence types (STs). The *K. pneumoniae* isolates were assigned to 225 known STs (Table S5). The most prevalent STs among *mcr-*positive *K. pneumoniae* isolates were ST15 (70/859, 8.15 %), which was detected in 14 countries across several hosts, ST11 (40/859, 4.66 %), which was found in 7 countries and several hosts, and ST147 (36/859, 4.19 %), which was detected in 10 countries from several hosts (Fig. 2). These results highlight the global dissemination of these resistance determinants across multiple genetic backgrounds. Insights into the evolutionary relationships among these genomes were obtained through pangenome analysis and phylogenetic reconstruction using the maximum likelihood method with 1,000 bootstrap replicates. The pangenome analysis categorized 1,238 genes as core (present in ≥99 % of genomes), 1,548 as soft core (present in ≥95 % but <99 %), 3,263 as shell (present in ≥15 % but <95 %), and 69,725 as cloud genes (present in <15 %) (Fig. 2). Furthermore, the analysis revealed that KP_WW21 likely resulted from clonal transmission, as evidenced by the presence of a small number of shared SNPs (<200 SNPs) across different countries, including the United Kingdom, Jordan, and Saudi Arabia (Table S6) (Li et al., 2023). This extensive genomic diversity is indicative of the adaptability of *mcr*-positive *K. pneumoniae* to diverse hosts and environments.

The genetic context of *mcr-*1.1 on pKP_WW21-mcr included *ISApl1*-*mcr-*1.1, with the *ISApl1* flanking only one side of the *mcr-*1.1 gene (Fig. S5). This pattern has been reported to enhance stability while reducing mobilization, which may contribute to the persistence of the plasmid in bacterial populations (Snesrud et al., 2018). The sequence of pKP_WW21-mcr was analyzed using BLASTN and 10 best matches (>90 % query coverage and >99 % identity) were identified, these include pSh487-m4 (KY363996.1), pC2 (LC473131.1), pSMEc189-mcr-1 (AP019686.1), pC1 (LC469775.1), pECJS-61–63 (KX254342.1), pSH12G950 (MH522410.1), pMCR-NMG21 (MK836306.1), pECJS61–63 (KX084393.1), pmcr-1 (CP047014.1), and PN21 (MG557851.1). In this regard, *mcr*-1 was identified in all retrieved plasmids, which were found in *S. sonnei* from China, *Salmonella enterica* from China and *E. coli* from Japan, China, and Thailand. Comparative analysis revealed a conserved backbone across these plasmids, while the surrounding regions of *mcr-*1 differed, particularly the presence of one-sided *ISApl1* in pKP_WW21-mcr (Fig. S5B). Conjugation experiments demonstrated that pKP_WW21-mcr was highly transferable under laboratory conditions, with a conjugation frequency of 1.4 × 10^–2^ CFU per recipient cell into *E. coli* J53. This transfer efficiency was notably higher than those reported in previous studies. The transconjugant exhibited >8-fold increase in the colistin MIC when compared with that of the recipeint cell (Table 1). These results suggested that the *mcr*-1.1 gene on pKP_WW21-mcr can be transferred and expressed in transconjugant.

### 3.5 Characterization of *tet*(X)-harboring plasmid

**Fig. 3:**
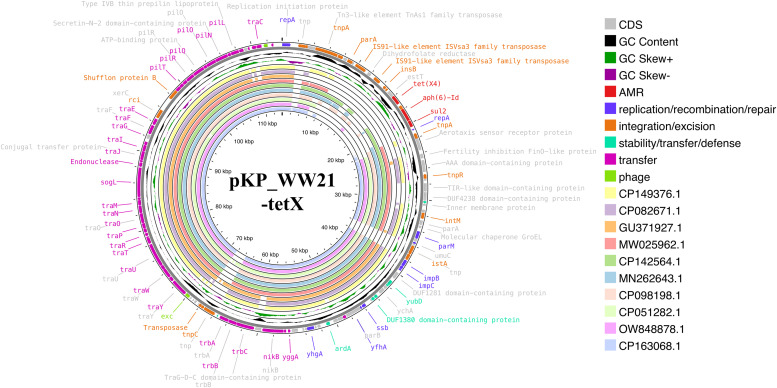
Genetic environment of *tet*(X4) in the pKP_WW21-tetX, an IncI1 plasmid. Circular comparison of pKP_WW21-tetX with ten other high similarity plasmids available in the NCBI database. The outermost circles indicate the plasmid pKP_WW21-tetX, with annotated genes shown.

A hybrid WGS approach revealed that *tet*(X) gene was located on a 113,859 bp IncI1-type plasmid (pKP_WW21-tetX; NCBI accession no CP192295). Notably, this is the first report of *tet*(X) being identified on an IncI1-type plasmid (Fig. 3). In addition to *tet*(X4), several other antimicrobial resistance genes were detected, including *aph(6)-Id, aph(3′')-Ib*, and *sul2*, which confer resistance to tetracyclines, aminoglycosides, and folate pathway antagonists, respectively. The presence of these resistance genes highlights the potential role of the plasmid in mediating multidrug resistance. Functional annotation of the plasmid identified 11 genes involved in replication/recombination/repair, 2 phage-associated genes, 21 genes linked to integration/excision, 7 genes related to stability/transfer/defense, and 45 genes exclusively involved in transfer activity. Importantly, a well-defined *tra* gene cluster was also present, which plays a crucial role in the conjugation process by facilitating horizontal gene transfer between bacterial cells. It has been reported that the capacity of the plasmid to disseminate antimicrobial resistance genes across bacterial populations is significantly enhanced by this cluster.

**Fig. 4:**
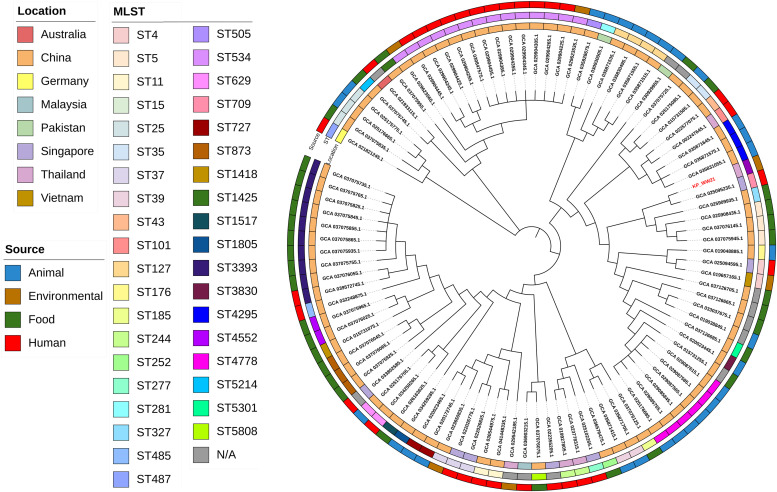
Phylogenetic tree of 99 *tet*(X)-positive *K. pneumoniae* isolates, with KP_WW21 from this study highlighted in red. The tree was constructed using maximum likelihood analysis based on 1238 core genome SNPs. From the innermost to the outermost ring, the colored bands indicate the location of isolation, MLST type, and source of isolation, respectively.

To investigate the prevalence of *tet(X)*-positive *K. pneumoniae*, genome sequences of 99 isolates were retrieved from the NCBI database (https://www.ncbi.nlm.nih.gov/pathogens/) by searching term “AMR_genotypes: *tet*(X)” and “species_taxid:573” (Table S7). Geographical distribution analysis revealed that China accounted for the majority of isolates (81/99, 81.82 %), followed by Singapore (7/99, 7.07 %) and Thailand (5/99, 5.05 %) (Fig. 1D). Host source analysis indicated that these isolates originated from diverse sources, including animals, food, humans, and environmental samples, underscoring the zoonotic and ecological reservoirs of *tet(X)*-positive strains. MLST analysis revealed significant genetic diversity among the 99 *tet(X)*-positive *K. pneumoniae* isolates, which were assigned to 39 known STs (Table S7). The most prevalent were ST534 (14/99, 14.14 %), detected in China from clinical sources. ST3393 (10/99, 10.10 %), identified in China across three different hosts and ST4778 (6/99, 6.06 %), found in China from two host types (Fig. 4). Pangenome analysis of the *tet(X)*-positive *K. pneumoniae* strains revealed significant genomic diversity. A total of *1,238* genes were identified as part of the core genome (present in ≥99 % of the genomes), while *1,548* genes constituted the soft core genome (present in ≥95 % but <99 %*)*. Additionally, *3,263* genes were classified as shell genes (present in ≥15 % but <95 %*),* and *69,725* genes were categorized as part of the cloud genome (present in <15 %*)*. However, the analysis revealed that KP_WW*21* had no clonal relationship with the global *tet*(X)-positive *K. pneumoniae* in the NCBI database, indicative of genetic diversity (Table S8). Thus, this distribution reflects the adaptability and genetic variability of *tet*(X)-positive *K. pneumoniae*, enabling survival and persistence across a range of environments and hosts.

**Fig. 5:**
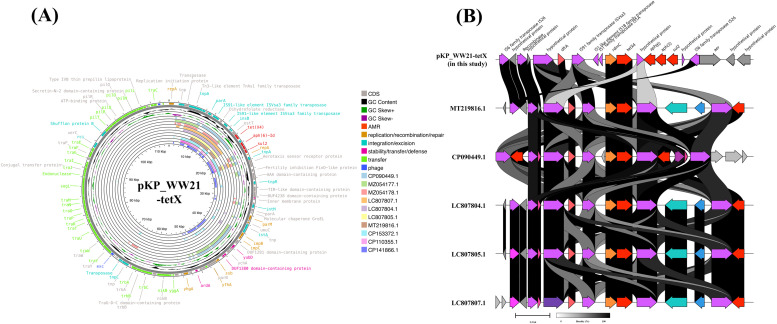
Genetic environment of *tet*(X4) on pKP_WW21-tetX, an IncI1 plasmid. (A) Circular comparison of pKP_WW21-tetX with ten other plasmids retrieved from the NCBI database, selected based on high sequence similarity to the composite transposon region of pKP_WW21-tetX. The outermost circle represents pKP_WW21-tetX, with annotated genes shown. (B) Linear alignment of the composite transposon carrying the *tet*(X4) gene on plasmid pKP_WW21-tetX with *tet*(X4)-carrying regions from five other plasmids retrieved from the NCBI database. The comparison illustrates the structural conservation of the transposon across plasmids.

The genetic context of *tet*(X4) on pKP_WW21-tetX included *IS3*-*IS3*-transposase-*TnAs1*-*IS16*-resolvase-*ISVsa3*-transposase-*estT*-*tet*(X4)-*aph6*-*aph3*-*sul2*. The sequence of pKP_WW21-tetX was then analyzed using BLASTN to identify related plasmids. The 10 best matches with query cover exceeding 60 % and sequence identity above 99 % were identified. These plasmids included pZ1323HSL0029–2 (CP149376.1), pN17S016–1 (CP082671.1), pEC_Bactec (GU371927.1), pX2–15 (MW025962.1), pA333_p0 (CP142564.1), pEC009.2 (MN262643.1), pZ0117EC0062–1 (CP098198.1), pST35–95,663.1B (CP051282.1), plasmidP2 (OW848878.1), and pW137_1 (CP163068.1).While these plasmids shared a conserved backbone region, the *tet*(X) gene was notably absent in all of them (Fig. 3). This suggests that the *tet*(X) gene in pKP_WW21-tetX may have been acquired through a distinct genetic event. Further analysis using MobileElementFinder revealed the presence of a putative composite transposon in pKP_WW21-tetX, indicating its potential for mobilization. To better understand the genetic context of *tet*(X4) in pKP_WW21-tetX, the region encompassing 8,776 bp upstream and 5,031 bp downstream of the gene was extracted and subjected to BLASTN analysis. The top 10 matches, with query cover exceeding 74 % and identity above 99 %, were identified. These included plasmids pPT62-tetX-108 kb (CP090449.1), pAB12–1-tetX4 (MZ054177.1), pHN13R-tetX4 (MZ054178.1), pD228 (LC807807.1), pD223 (LC807804.1), pD225 (LC807805.1), pRF173–1_87k_tetX (MT219816.1), pKPNB_2008_54,824.1 (CP153372.1), pECC2783_a (CP110355.1), and plasmid *E. coli* strain numer 3 (CP141866.1). Notably, as shown in Fig. 5A, the *tet*(X) gene was identified in only 5 of these plasmids, which were primarily associated with *E. coli* isolates from China and Thailand. The Chinese isolates originated from pigeons, while the Thai isolates were recovered from pigs. A detailed comparison of the sequence of pKP_WW21-tetX with these related plasmids revealed both similarities and distinctions in their genetic architecture. While all shared a conserved backbone region, variations in the surrounding regions of *tet*(X) were observed (Fig. 5B). This variability may influence the stability and mobility of the *tet*(X) gene across different plasmids. Conjugation experiments demonstrated that pKP_WW21-tetX was highly transferable under laboratory conditions, with a conjugation frequency of 2.7 × 10^–2^ CFU per recipient cell into *E. coli* J53. The transconjugant exhibited a 32-fold increase in the tigecycline MIC when compared with that of the recipeint cell (Table 1). These results suggest that the *tet*(X4) gene is transferable and funtionally expressed in the transconjugant.

## 4 Discussion

In this study, we isolated a multidrug-resistant strain of *K. pneumoniae* KP_WW21, from wastewater collected at a pet grooming shop. Hybrid whole-genome sequencing confirmed that the isolate belongs to the ST14 lineage and harbors four separate plasmids carrying *bla*_NDM-1_, *bla*_OXA-232_, *mcr*-1.1, and *tet*(X4). ST14 is increasingly recognized as a high-risk, globally disseminated clone associated with extensive drug resistance and significant clinical impact. Although it has not traditionally been classified among the most dominant high-risk lineages, such as ST258 or ST147, ST14 has consistently demonstrated the ability to acquire and maintain diverse AMR genes, including *bla*_NDM_, *bla*_OXA-48-like_, and *mcr*-1 (David et al., 2019; Wyres et al., 2020). Notably, ST14 is among the most frequently reported *bla*_NDM_-positive *K. pneumoniae* lineages and has been identified across multiple continents (Wu et al., 2019). Most ST14 strains have been recovered from human clinical specimens and often produce carbapenemases such as *bla*_NDM_, *bla*_KPC_, *bla*_OXA-48-like_, either alone or in combination (Al‑Baloushi et al., 2018; Moubareck et al., 2018). The convergence of carbapenemase production, multidrug resistance, and a high potential for horizontal gene transfer underscores the serious public health threat posed by this lineage. While the presence of *mcr-*1 in ST14 has been previously reported, to our knowledge, this is the first report of a plasmid-borne *tet*(X4) gene in an ST14 isolate, thereby expanding the resistance repertoire of this emerging clone.

Comparative genomic analysis revealed that *K. pneumoniae* KP_WW21 shares key resistance determinants with other *K. pneumoniae* strains harboring *mcr-*1, *tet*(X4), *bla*_NDM-1_, and *bla*_OXA-232_. The identification of *bla*_NDM-1_ on a hybrid *IncFIB*-*IncHI1B* plasmid highlights the role of hybrid plasmids in the dissemination of AMR genes. This plasmid harbors multiple resistance genes and exhibits a composite transposon structure flanked by *ISAba125* and *ISEc33*, highlighting its potential for horizontal gene transfer and integration into various genomic contexts. The presence of *ISEc33* further enhances the mobility of *bla*_NDM-1_, facilitating its dissemination among bacterial populations. The conserved genetic module surrounding *bla*_NDM-1_ suggests functional optimization for persistence and horizontal transfer (Acman et al., 2022).

The *bla*_OXA-232_ gene was located on a ColKP3-type plasmid characterized by a minimal set of replication, defense, and transfer-associated genes, consistent with its classification as non-conjugative plasmid. This plasmid type has been reported predominantly in Thailand, China, India, and the USA, indicating regionally influenced dissemination. Comparative analysis with the reference plasmid pkNICU5 revealed a high degree of conservation. As expected, the ColKP3 plasmid was not transferable under selection with ertapenem or tigecycline (Potron et al., 2013; Yin et al., 2017). However, successful plasmid transfer was observed when colistin was used as the selective agent, indicating that antibiotic pressure may promote the mobilization of plasmids that are otherwise non-transmissible. Several potential mechanisms could explain this unexpected transfer. Colistin is known to disrupt bacterial outer membrane integrity, which may facilitate plasmid uptake or enhance cell-to-cell contact (Ding et al., 2022; Kim et al., 2014). Additionally, colistin exposure can induce the bacterial SOS response, potentially activating latent mobility pathways (Aminov 2011; Hastings et al., 2004). Another possible explanation is the presence of conjugative helper plasmids within the donor strain that supply missing transfer functions required for mobilization of small plasmids (Chen et al., 2021). These mechanisms, individually or synergistically, may have enabled the transfer of the ColKP3 plasmid despite its non-conjugative nature (Bengtsson‑Palme et al., 2018). In our study, we identified the presence of a co-transferred plasmid, pKP_WW21-mcr, which harbored a VirBR regulatory cluster. This transcriptional regulator system has recently been reported to enhance conjugative transfer of IncX3 plasmids carrying *bla*_NDM_ genes by counter-silencing negative regulators of plasmid transmission (Gao et al., 2025; Ma et al., 2024). While VirBR has been primarily studied in IncX3-type plasmids, its presence on pKP_WW21-mcr suggests a broader role in facilitating conjugation, potentially by modulating host regulatory pathways or enhancing plasmid stability during horizontal gene transfer. Thus, the mobilization of the ColKP3 plasmid in our study may have been indirectly supported by such regulatory elements on co-resident conjugative plasmids. Interestingly, hybrid WGS of the transconjugant revealed that the ColKP3 plasmid increased in size from 6.1 kb to 12.3 kb. This suggests possible genetic fusion with co-resident plasmids or the acquisition of mobile genetic elements, such as insertion sequences or integrons. Such recombination events have been well documented in Enterobacterales and are thought to enhance plasmid stability, expand host range, or provide functional complementation (Partridge et al., 2018). These findings are supported by previous works showing that even small, non-conjugative plasmids like ColKP3 can become mobilized when co-existing with large, conjugative plasmids that supply missing transfer functions (Chen et al., 2021). Overall, these findings challenge the assumption that non-conjugative plasmids are static and emphasize the importance of antibiotic-induced selective pressure in shaping plasmid mobility and resistance gene dissemination.

For *mcr*-1, the plasmid pKP_WW21-mcr belongs to the IncI2 replicon type, which is the dominant carrier of *mcr*-1 in Asia (Matamoros et al., 2017). It contains a full set of transfer-associated genes, indicating strong potential for conjugative transfer and stable maintenance. The *mcr*-1.1 gene is positioned next to a single copy of *ISApl1* on one side, forming the *ISApl1*–*mcr*-1.1 configuration. This partial flanking is believed to enhance plasmid stability while limiting mobilization, which may contribute to its long-term persistence in bacterial populations (Snesrud et al., 2018). MLST and pangenome analyses further revealed the extensive genetic diversity of *mcr*-positive *K. pneumoniae*, highlighting their adaptability to various hosts and environments. Clonal transmission events, evidenced by shared SNPs among isolates from diverse countries, underscore the global dissemination of these resistance determinants. Furthermore, the identification of *mcr*-positive isolates in both human and non-human reservoirs emphasizes the zoonotic and environmental origins of these pathogens, necessitating comprehensive surveillance and intervention efforts. The comparative genomic analysis of pKP_WW21-mcr and related plasmids revealed a conserved backbone with diverse surrounding regions, indicating evolutionary pressures shaping their persistence and dissemination. The high transfer efficiency and stability of pKP_WW21-mcr in laboratory settings suggest that it plays a significant role in the horizontal transfer of *mcr*-1.1, further complicating efforts to control antimicrobial resistance.

For *tet*(X4), pKP_WW21-tetX is the first known IncI1-type plasmid carrying this gene, expanding the known range of plasmid backbones associated with tigecycline resistance (IncX1, IncFII, IncQ1, IncN, and hybrid plasmid types) (Gao et al., 2022; Sun et al., 2020; Zhang et al., 2022). The plasmid also encodes *aph(6)-Id, aph(3′')-Ib*, and *sul2*, further contributing to its multidrug resistance profile. The presence of a complete *tra* operon and a composite transposon supports its high conjugation potential and mobility. Geographically, most *tet*(X)-positive *K. pneumoniae* isolates have been reported from China, with fewer from Singapore and Thailand (Fig. 1D). Host analysis suggests both zoonotic and environmental reservoirs, supporting the role of diverse ecosystems in maintaining these resistance determinants. The genetic diversity observed through MLST and pangenome analysis reflects the adaptability of these strains, enabling their persistence in various ecological niches. The conserved backbone region among pKP_WW21-tetX and related plasmids, coupled with the absence of the *tet*(X) gene in most related plasmids, suggests that *tet*(X) was acquired through a distinct genetic event. The presence of a composite transposon further highlights the potential of plasmid for mobilization, which may facilitate the dissemination of *tet*(X) mediated resistance. The association of *tet*(X4) with IncI1 in this study is unprecedented, expanding the known replicon diversity of *tet*(X) harboring plasmids. These findings warrant further investigation into the mechanisms underlying plasmid stability, transfer efficiency, and genetic variability, which are critical for understanding the dissemination of *tet*(X) positive *K. pneumoniae*.

While our conjugation assays provided important evidence of plasmid transfer and antibiotic-driven mobilization under laboratory conditions, it is important to note that these experiments may not fully replicate the complex and variable conditions present in natural environments. Factors such as microbial community interactions, environmental stresses, nutrient availability, and host factors can significantly influence horizontal gene transfer dynamics in situ. Therefore, the rates and mechanisms of plasmid transfer observed in this study should be interpreted with caution when extrapolating to real-world settings. Additionally, environmental metadata such as temperature, pH, organic matter content, or seasonal variation were not recorded at the time of sampling. Each site was sampled only once, and the study was not designed to capture longitudinal or seasonal trends. As such, we were unable to assess the potential influence of environmental parameters on the persistence or dissemination of AMR genes. While this limits our ability to correlate resistance profiles with site-specific environmental factors, the primary objective of the study was to investigate the presence and genetic characteristics of antimicrobial-resistant bacteria in wastewater from pet grooming facilities. Future studies incorporating systematic environmental monitoring and time series sampling would be valuable for understanding the ecological drivers of resistance gene distribution in such settings.

Together, our findings offer comprehensive insight into the genomic architecture and mobility of resistance genes in *K. pneumoniae* KP_WW21, a wastewater-derived ST14 strain co-harboring *bla*_NDM-1_, *bla*_OXA-232_, *mcr*-1.1, and *tet*(X4) on four distinct plasmids. The detection of ST14 in an environmental source is particularly concerning, as this lineage has largely been associated with human infections. Its presence in wastewater raises the possibility of interspecies and environmental transmission, underscoring the urgent need for integrated surveillance. Importantly, this study demonstrates the mobilization of a ColKP3-type *bla*_OXA-232_ plasmid under colistin pressure, highlighting a mechanism by which non-conjugative plasmids may acquire transferability. Additionally, the identification of *tet*(X4) on an IncI1 plasmid represents a novel replicon association, with strong implications for the expanding mobility of tigecycline resistance.

In conclusion, KP_WW21 exemplifies the convergence of a high-risk clone with highly mobile, resistance-bearing plasmids in a non-clinical setting. The ability of selective pressures such as colistin to drive the mobilization of even non-conjugative plasmids presents a significant risk for the spread of AMR. These findings reinforce the critical importance of One Health-based AMR strategies that integrate environmental monitoring, plasmid surveillance, and antimicrobial stewardship to address the evolving landscape of resistance dissemination.

## CRediT authorship contribution statement

**Thanawat Phuadraksa:** Conceptualization, Data curation, Formal analysis, Funding acquisition, Investigation, Methodology, Visualization, Writing – original draft, Writing – review & editing. **Yanisa Choominthong:** Formal analysis, Investigation. **Sineewanlaya Wichit:** Formal analysis, Methodology. **Sakda Yainoy:** Conceptualization, Formal analysis, Funding acquisition, Investigation, Methodology, Visualization, Resources, Supervision, Project administration, Writing – review & editing.

## Declaration of competing interest

The authors declare that they have no known competing financial interests or personal relationships that could have appeared to influence the work reported in this paper.

## Data Availability

The datasets generated during the current study are available in the National Center for Biotechnology Information repository (NCBI) repository under BioProject accession number PRJNA1265403, with GenBank accession numbers CP192293–CP192298, and CP195071.

## Appendix Supplementary materials

Image, application 1

Image, application 2

Image, application 3

Image, application 4

Image, application 5

Image, application 6

Image, application 7

Image, application 8

Image, application 9

Image, application 10

Image, application 11

Image, application 12

Image, application 13
