# Supplementary figures and images for: Emergence of Klebsiella pneumoniae ST14 co-harboring blaNDM-1, blaOXA-232, mcr-1.1, and a novel IncI1 tet(X4) plasmid, with evidence of ColKP3 mobilization under antibiotic pressure
Source: Curr Res Microb Sci. 2025 Aug 28;9:100466. doi: 10.1016/j.crmicr.2025.100466 (PMC12444185; doi:10.1016/j.crmicr.2025.100466)

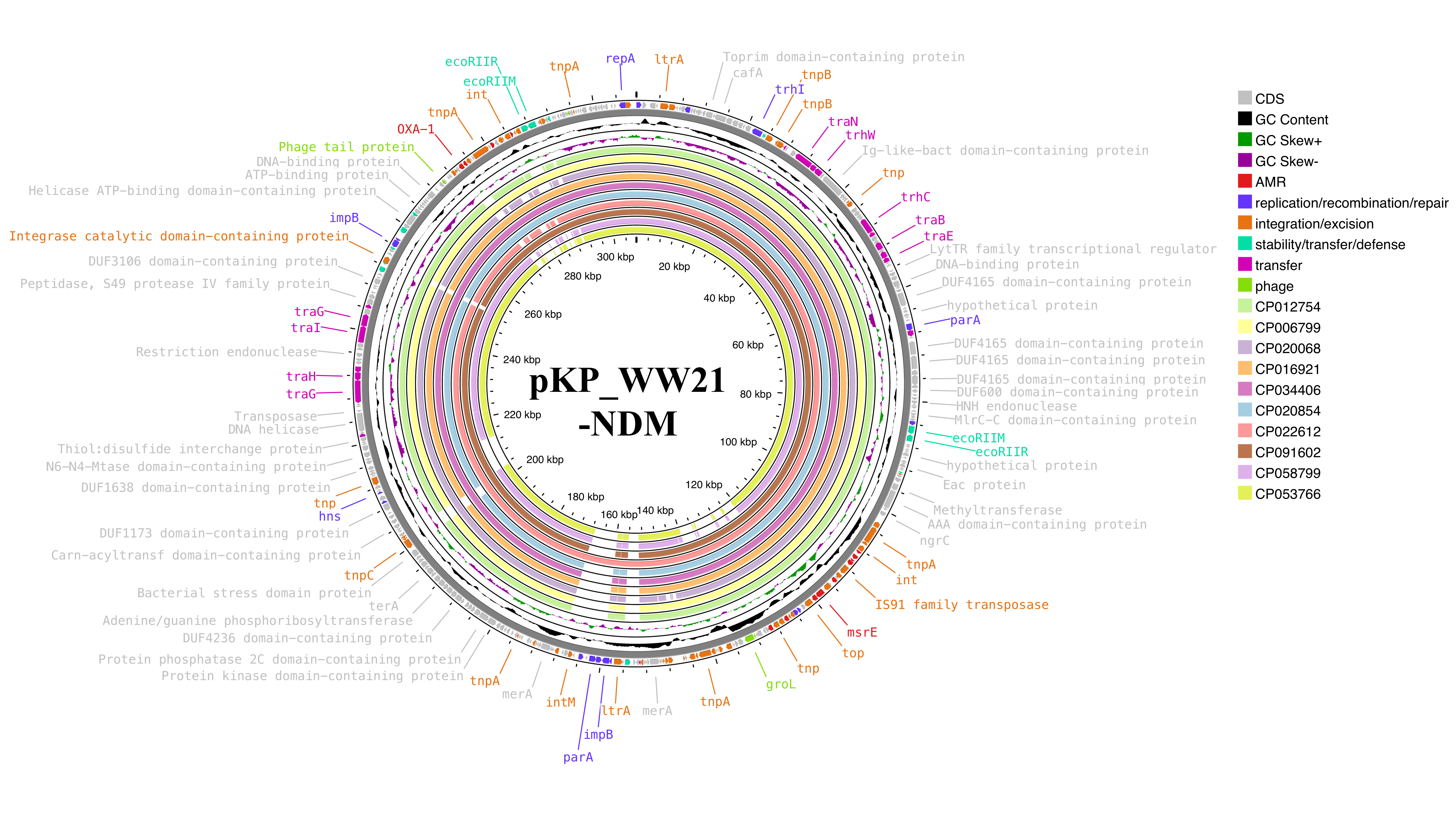

Supplement: Supplementary file 1 [file mmc1.zip › mmc1.TIF]

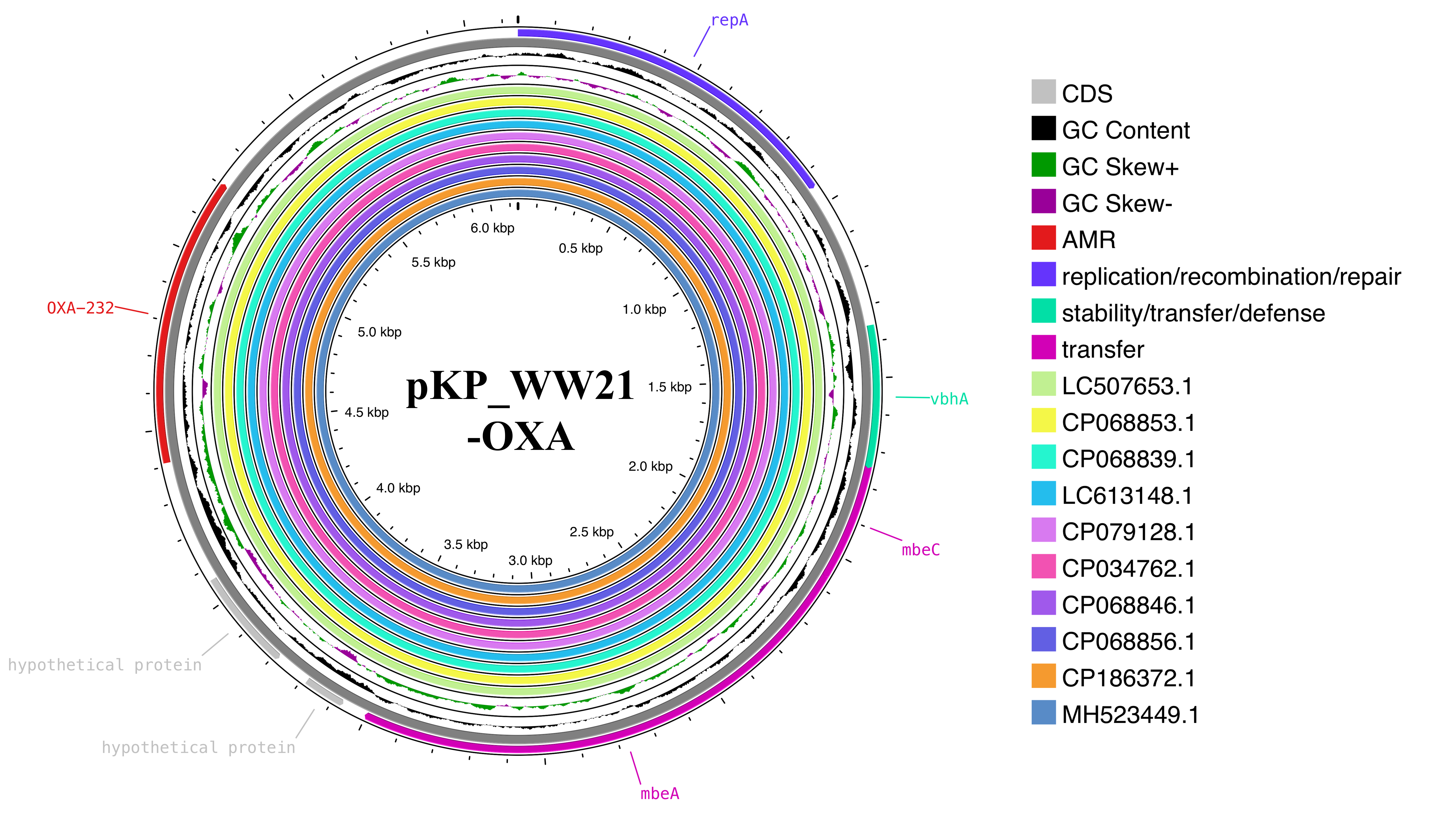

Supplement: Supplementary file 2 [file mmc2.zip › mmc2.TIF]

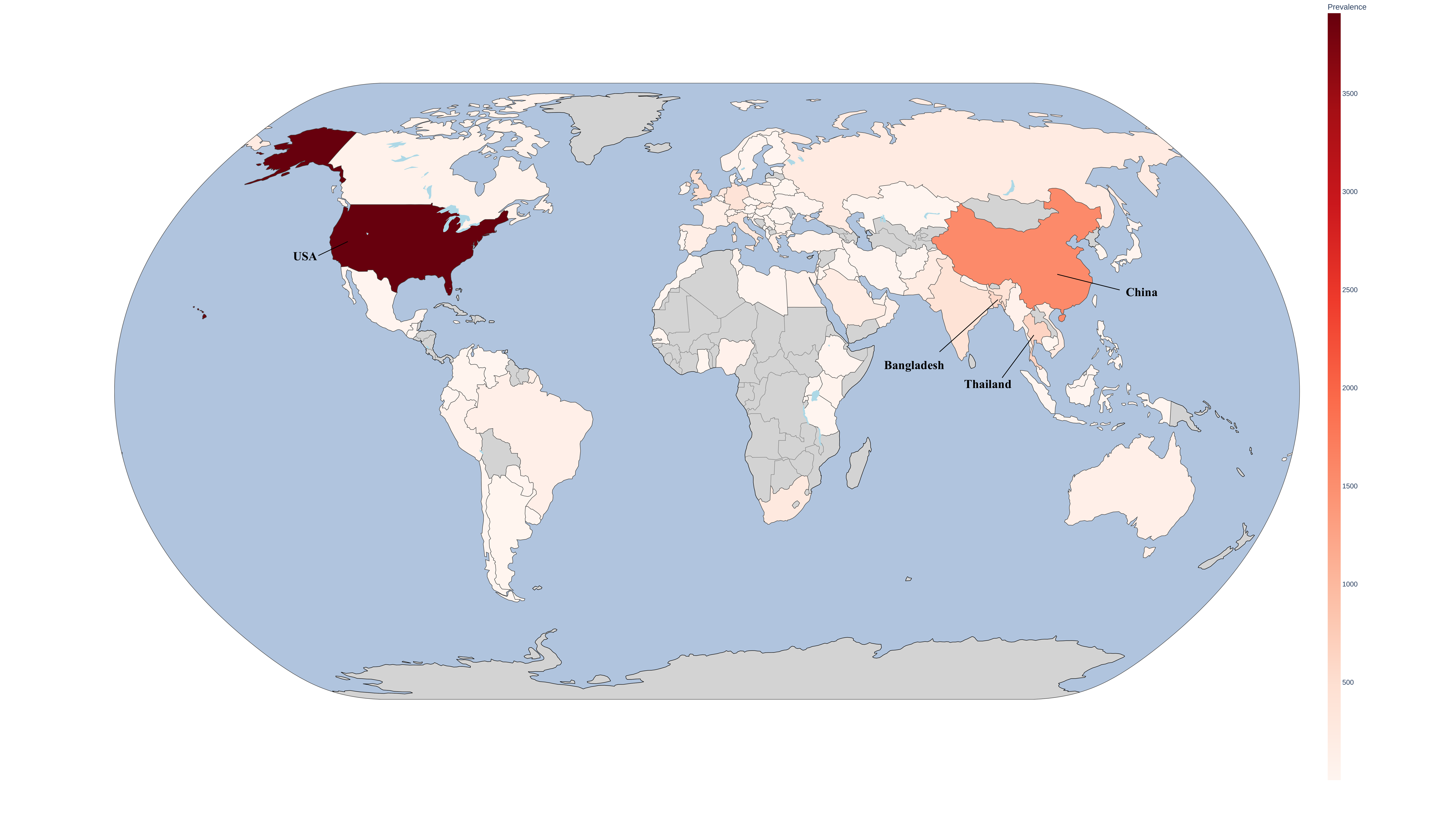

Supplement: Supplementary file 3 [file mmc3.zip › mmc3.TIF]

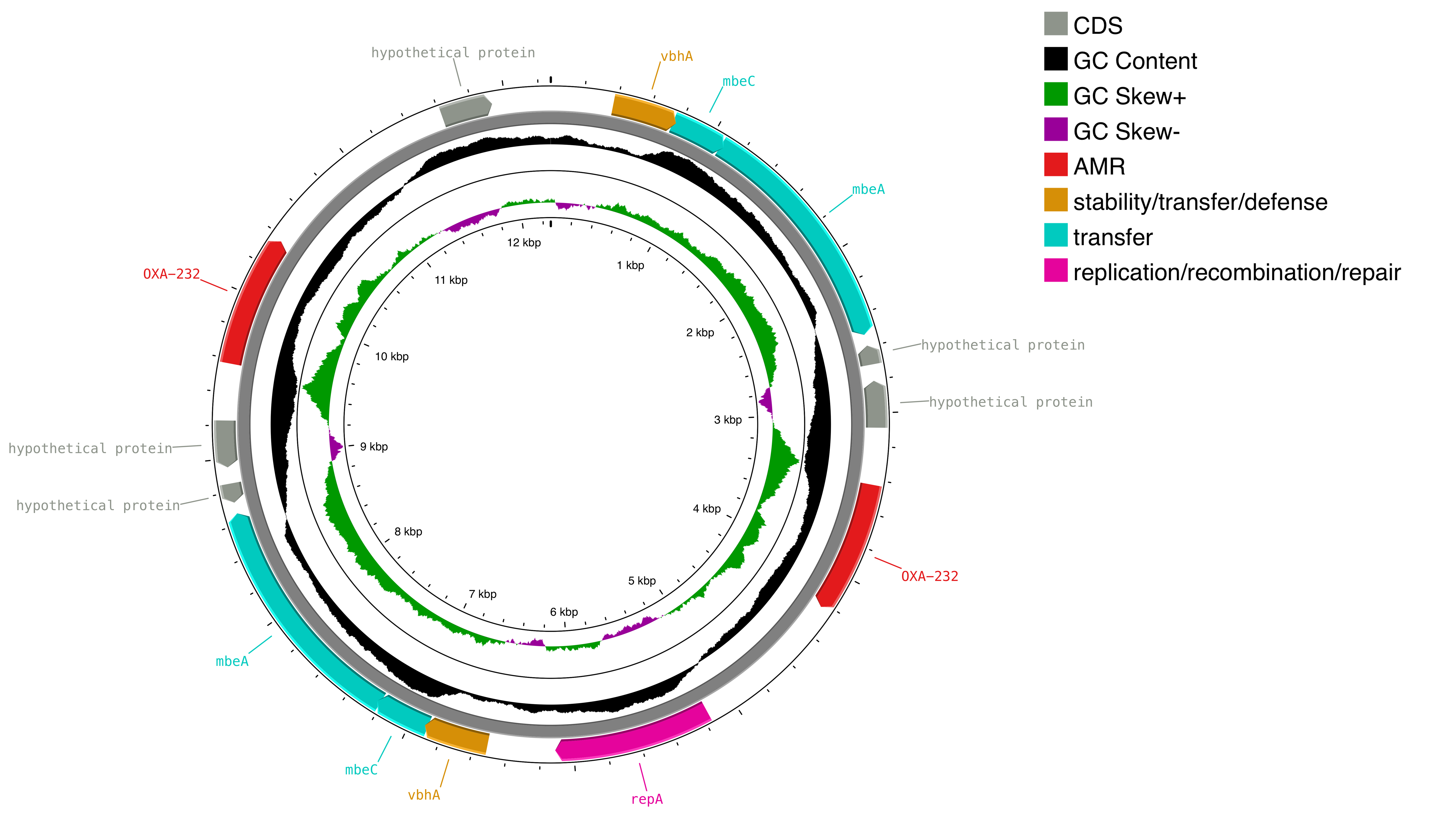

Supplement: Supplementary file 4 [file mmc4.zip › mmc4.TIF]

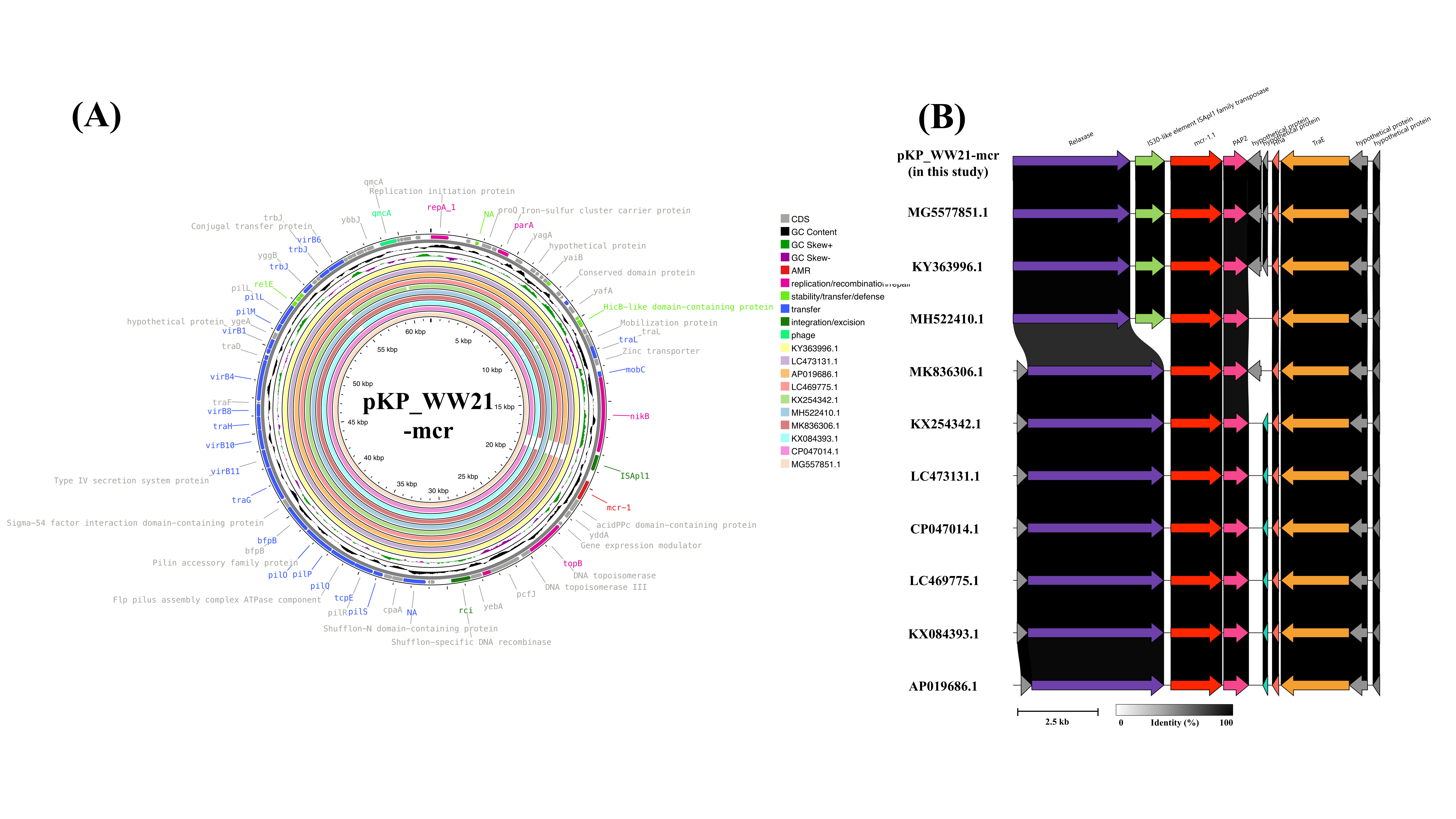

Supplement: Supplementary file 5 [file mmc5.zip › mmc5.TIF]
