# Supplementary material for: Emergence of Klebsiella pneumoniae ST14 co-harboring blaNDM-1, blaOXA-232, mcr-1.1, and a novel IncI1 tet(X4) plasmid, with evidence of ColKP3 mobilization under antibiotic pressure
Source: Curr Res Microb Sci. 2025 Aug 28;9:100466. doi: 10.1016/j.crmicr.2025.100466 (PMC12444185; doi:10.1016/j.crmicr.2025.100466)
Supplement: Supplementary file 6 [file mmc6.docx]

Table S1. Feature of the isolate

| DNA types | DNA size (bp) | MLST/plasmid replicon | AMR gene | Accession no. |
| --- | --- | --- | --- | --- |
| KP_WW21-chromosome | 5,362,511 | ST 14 | *aac(6')-Ib-cr*, *bla*CTX-M-15, *bla*SHV-106, *bla*SHV-28, *bla*OXA-1, *fosA6*, *catB3*, *OqxA*, *OqxB*, and *dfrA1* | CP192293 |
| pKP_ WW21-NDM | 307,479 | *IncFIB(Mar)/ IncHI1B/IncR* | *aadA2*, *bla*_OXA-1_, *bla*_NDM-1_, *msr(E)*, *aph(3')-VI*, *armA*, *qnrB1*, *sul1*, *dfrA12*, and *dfrA14* | CP192294 |
| pKP_ WW21-OXA | 6,141 | *ColKP3* | *bla*_OXA-232_ | CP192298 |
| pKP_ WW21-mcr | 61,805 | *IncI2* | *mcr*-1.1 | CP192297 |
| pKP_ WW21-tetX | 113,859 | *IncI1* | *tet*(X4), *aph(6)-Id*,  *aph(3'')-Ib*, and *sul2* | CP192295 |
| pKP_ WW21-5 | 105,457 | *IncFIB(K)* | - | CP192296 |
